# MgCa-Based Alloys Modified with Zn- and Ga-Doped CaP Coatings Lead to Controlled Degradation and Enhanced Bone Formation in a Sheep Cranium Defect Model

**DOI:** 10.1021/acsbiomaterials.4c00358

**Published:** 2024-06-14

**Authors:** Seyda Gokyer, Yanad Abou Monsef, Senem Buyuksungur, Jurgen Schmidt, Alina Vladescu Dragomir, Sencer Uygur, Cagdas Oto, Kaan Orhan, Vasif Hasirci, Nesrin Hasirci, Pinar Yilgor

**Affiliations:** † Department of Biomedical Engineering, Ankara University, Ankara 06830, Turkey; ‡ Anatomic Pathology Department, National Veterinary School of Toulouse, Toulouse 31300, France; § BIOMATEN, Center of Excellence in Biomaterials and Tissue Engineering, Middle East Technical University (METU), Ankara 06800, Turkey; ∥ Gruppenleiter Elektrochemie, Prüssingstraße 27b, INNOVENT e.V. Technologieentwicklung, Jena 07745, Germany; ⊥ 409 Atomistilor St., National Institute of R&D for Optoelectronics—INOE 2000, Magurele 77125, Romania; # Research School of Chemistry & Applied Biomedical Sciences, National Research Tomsk Polytechnic University, Tomsk 634050, Russia; ^∇^Faculty of Veterinary Medicine Department of Surgery, ^○^Faculty of Veterinary Medicine Department of Anatomy, ^◆^Medical Design Research and Application Center MEDITAM, ^¶^Faculty of Dentistry Department of Dentomaxillofacial Radiology, Ankara University, Ankara 06100, Turkey; ^††^Department of Biomedical Engineering, ^‡‡^Graduate Department of Biomaterials, ^§§^Biomaterials Center, Acibadem Mehmet Ali Aydinlar University, Istanbul 34752, Turkey; ∥∥ METU Department of Chemistry, Ankara 06800, Turkey; ⊥⊥ Near East University Tissue Engineering and Biomaterials Research Center, Nicosia 99138, TRNC Mersin 10, Turkey

**Keywords:** biodegradable metal, Mg-based alloy, bone regeneration, sheep model, *in vivo*, neovascularization

## Abstract

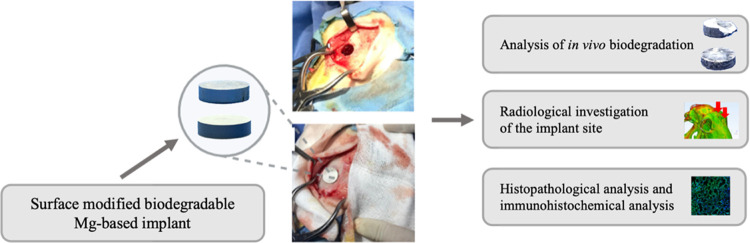

Mg-based biodegradable metallic implants are gaining increased attraction for applications in orthopedics and dentistry. However, their current applications are hampered by their high rate of corrosion, degradation, and rapid release of ions and gas bubbles into the physiological medium. The aim of the present study is to investigate the osteogenic and angiogenic potential of coated Mg-based implants in a sheep cranial defect model. Although their osteogenic potential was studied to some extent, their potential to regenerate vascularized bone formation was not studied in detail. We have studied the potential of magnesium–calcium (MgCa)-based alloys modified with zinc (Zn)- or gallium (Ga)-doped calcium phosphate (CaP) coatings as a strategy to control their degradation rate while enhancing bone regeneration capacity. MgCa and its implants with CaP coatings (MgCa/CaP) as undoped or as doped with Zn or Ga (MgCa/CaP + Zn and MgCa/CaP + Ga, respectively) were implanted in bone defects created in the sheep cranium. MgCa implants degraded faster than the others at 4 weeks postop and the weight loss was ca. 50%, while it was ca. 15% for MgCa/CaP and <10% in the presence of Zn and Ga with CaP coating. Scanning electron microscopy (SEM) analysis of the implant surfaces also revealed that the MgCa implants had the largest degree of structural breakdown of all the groups. Radiological evaluation revealed that surface modification with CaP to the MgCa implants induced better bone regeneration within the defects as well as the enhancement of bone–implant surface integration. Bone volume (%) within the defect was ca. 25% in the case of MgCa/CaP + Ga, while it was around 15% for undoped MgCa group upon micro-CT evaluation. This >1.5-fold increase in bone regeneration for MgCa/CaP + Ga implant was also observed in the histopathological examination of the H&E- and Masson’s trichrome-stained sections. Immunohistochemical analysis of the bone regeneration (antiosteopontin) and neovascularization (anti-CD31) at the defect sites revealed >2-fold increase in the expression of the markers in both Ga- and Zn-doped, CaP-coated implants. Zn-doped implants further presented low inflammatory reaction, notable bone regeneration, and neovascularization among all the implant groups. These findings indicated that Ga- and Zn-doped CaP coating is an important strategy to control the degradation rate as well as to achieve enhanced bone regeneration capacity of the implants made of Mg-based alloys.

## 1 Introduction

Bone fractures are among the most common complications, necessitating creative solutions for long-lasting and efficient tissue healing. In many applications, new materials such as polymers, ceramics, and their novel composites are gradually replacing metals; however, metallic implants are still the favored option for orthopedic and dental purposes.^1−3^ Their exceptional mechanical strength, which is especially important in correcting abnormalities in load-bearing bones, is the reason for this preference. Biodegradable implants have become a viable substitute for conventional metallic implants in recent decades, providing a distinct combination of biocompatibility and mechanical strength. Researchers have focused on biodegradable metals in an effort to avoid the need for a secondary surgery to remove metallic implants after the defects heal or when a pediatric patient outgrows the implant. These metals are known for their capacity to deteriorate gradually in the physiological milieu while retaining sufficient strength until the process of tissue regeneration is completed. Alloys based on magnesium (Mg) have attracted the most attention due to their exceptional qualities that make them ideal for use in bone regeneration and fracture healing.^1−7^

In the physiological environment, pure Mg has a high rate of corrosion and fast ion and hydrogen gas release.^7,8^ On the other hand, Mg-based alloys containing aluminum (Al), calcium (Ca), strontium (Sr), manganese (Mn), zirconium (Zr), tin (Sn), and zinc (Zn) can regulate the quick release of hydrogen gas and boost corrosion resistance.^9,10^ Novel Mg alloys can be created for medical use by adjusting their microstructure, mechanical strength, osteogenic qualities, degradation rates, and degradation products.^11−13^ Recent studies have shown that surface modification strategies can be used to improve the aforementioned properties of Mg-based alloys.^7^ These adjustments include adding different metal coatings to the alloy’s surface or changing its surface structure by adding pores, nanodesigns, or crystals. These developments highlight the versatility and reactivity of Mg alloys to diverse biomedical needs, while also expanding their possible uses in the realm of medicine.^14,15^ Furthermore, enhancing the corrosion resistance of Mg-based alloys can be achieved by coating them with protective compounds like hydroxyapatite (HAp) or other biocompatible substances.^4,6,16−19^ Furthermore, by carefully planning their release of ions, these coatings can aid in bone regeneration and enhance the overall biocompatibility of the implant. In the meantime, some macromolecules, like lipids or proteins, might be adhered to the surface and delay decomposition while also encouraging better cell adhesion and attachment stability.^20,21^ Protein adsorption results in enhanced cell adhesion, proliferation, and extracellular matrix (ECM) deposition, all of which promote tissue regeneration by increasing the cell affinity for the metal surface. The implant’s microenvironment experiences a change in physiological homeostasis as a result of these combined responses.^16,18,22^

Biodegradable implants made of Mg-based alloys are frequently coated with hydroxyapatite or other biocompatible materials to increase their corrosion resistance using a variety of techniques.^23^ It is possible to intentionally design these coatings to release ions, which promote bone repair. The addition of Ca by alloying makes it possible to modify the degrading behavior of Mg-based implants to a desired degree for certain *in vivo* uses.^24,25^ Changes in the rate of degradation are directly related to the amount of Ca in the alloy. The *in vivo* performance of Mg–Ca binary alloys is improved by the inclusion of other components. Notably, improvements in mechanical strength, antimicrobial efficacy, and degrading qualities have been linked to the addition of zinc (Zn) to Mg–Ca binary alloys.^26,27^ Gallium (Ga) addition enhanced corrosion resistance and had a favorable effect on osteoblast development and bone–implant bonding.^11,12,18,26,28,29^

Recent studies have focused on modifying Mg alloys to promote angiogenic responses, with the aim of optimizing their performance in implantable devices. Strategies include the incorporation of specific alloying elements, such as strontium or zinc, which have been shown to positively influence vascularization. Additionally, surface modifications and coatings are explored to create a microenvironment conducive to angiogenesis, fostering interactions between the implant and surrounding tissues.^12,30−34^ In our previous work, we reported the preparation of MgCa implants coated with Zn- or Ga-doped CaP as well as their chemical, physical, mechanical, and *in vitro* characterization in detail.^26,28^ This study is concentrated on the *in vivo* application of previously characterized implant materials in a sheep cranial implantation model.

In this work, using a sheep cranium defect model, we examined the effects of these modified materials on bone tissue regeneration. Microarc oxidation was used to modify the surfaces of MgCa alloys (MgCa) by applying CaP coatings (MgCa/CaP) that were either undoped or doped with Zn or Ga (MgCa/CaP + Zn and MgCa/CaP + Ga, respectively).^26,28^ We have implanted these materials into the bone defects created in the sheep skull. Animals were sacrificed after 4 weeks, and biodegradation of the implants as well as the bone regeneration and neovascularization within the defects were studied. Biodegradation was assessed gravimetrically and morphologically by scanning electron microscopy (SEM). Histopathological analysis was used to evaluate the bone–implant interface and the extent of bone regeneration within the defect by H&E and Masson’s trichrome staining. Moreover, immunohistochemistry was performed to assess osteogenesis and neovascularization within the defect by immunofluorescence staining for osteopontin (OPN) and CD31, respectively. This study is novel in terms of assessment of vascularized bone (osteogenic and angiogenic) regeneration potential of CaP coatings on MgCa implants doped with Zn and Ga in a sheep cranial implantation model.

## 2 Experimental Section

### 2.1 Bone Defect Model and Implantation

**Figure 1:**
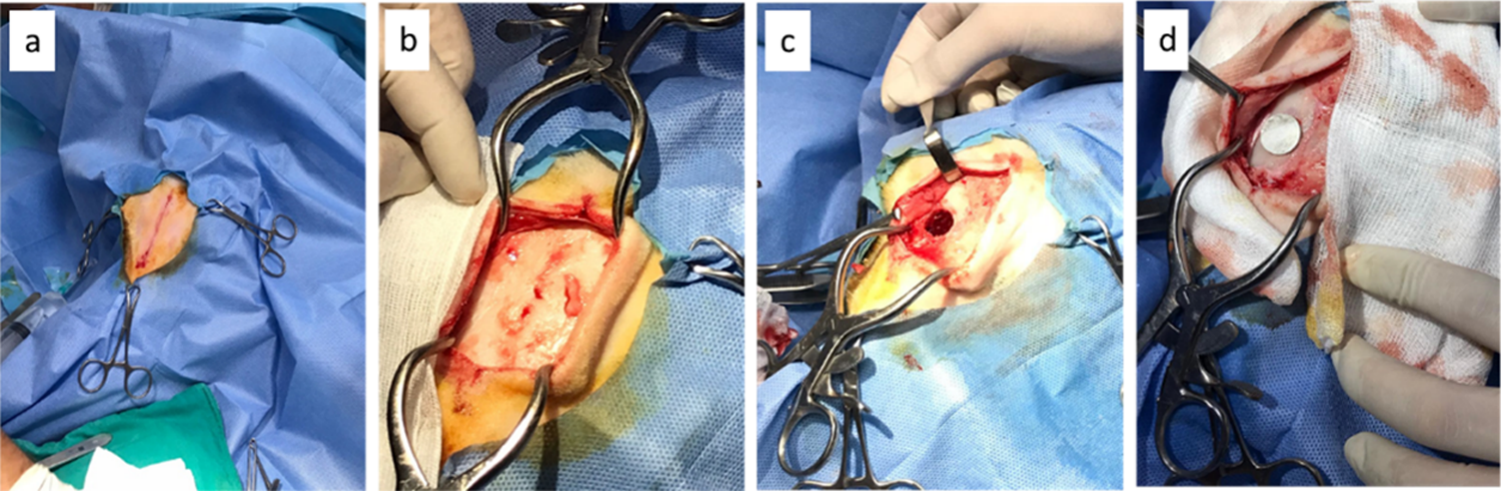
Implantation of MgCa-based implants within the sheep cranium defect model. (a, b) Surgery for the exposure of the sheep cranium; (c, d) implantation procedure.

Biodegradable implants made of MgCa alloys were prepared and surface modified with CaP coatings containing either Zn or Ga using microarc oxidation, as we previously described.^26,28^ To confirm the bone regeneration potential of the implants, critically sized circular skull defects (ε1.5 cm in diameter) were created in 16 adult (1-year-old), healthy male sheep. Sheep were randomly divided into 4 groups for evaluation, and the following implants were applied: (1) MgCa, (2) MgCa modified with CaP (MgCa/CaP), (3) MgCa modified with CaP containing Zn (MgCa/CaP + Zn), and (4) MgCa modified with CaP containing Ga (MgCa/CaP + Ga). Animal procedures were performed at the Ankara University Faculty of Veterinary Medicine, Experimental and Applied Research Farm, in accordance with a protocol approved by the Ankara University Animal Research Ethics Committee (2021-10-77). The sheep were anesthetized with i.m. xylazine (0.2 mg/kg) + ketamine HCL (20 mg/kg) combination after atropine injection (0.2 mg/kg). The scalp of the sheep was shaved, and the surgical site was disinfected. A skin incision along the sagittal line on the top portion of the skull was made, and the periosteum was also elevated carefully to reveal the skull. Circular defects of 4 mm depth were created by using trephine burr with continuous saline irrigation on the parietal bone (Figure 1). After the implants were placed within the defects, the periosteum, subcutaneous fascia, and skin of the skull were sutured, respectively. In the postoperative period, a drain was placed for the first 3 days, and nonsteroidal anti-inflammatory agents were administered. All animals were fed ad libitum and were euthanized by an overdose of sodium pentothal at 4 weeks after implantation. The bone samples were collected from the operation site and were fixed for 48 h in 10% neutral buffered formalin before micro-CT, histopathological, and immunohistochemical analysis.

### 2.2 *In Vivo* Biodegradation Analysis

Implants were excised carefully at 4 weeks after implantation, following scarification of the animals. Implants were investigated for *in vivo* biodegradation behavior. The deterioration of the implant surface was observed by SEM (Quanta 400F Field Emission SEM). For this, the implants were carefully collected from the bone defect site, rinsed in 1,4-piperazinediethanesulfonic acid (PIPES) buffer, dehydrated in a series of ethanol, and sputter coated with Au–Pd (15 nm). Observations were carried out at 20 kV, and images were recorded at low magnifications (30–5000×).

The percent weight loss of the implants was calculated according to the following equation:
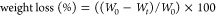
where *W*_0_ and *W*_t_ are the dry weights of the sample before and after 4 weeks of implantation, respectively.

### 2.3 Cone Beam and Micro-Computed Tomography

The whole sheep cranium with defects was first scanned with 3D Planmeca Promax Max (Planmeca Oy, Helsinki, Finland) cone beam computer tomography (CBCT) in order to visualize the implant site. The images were taken as DICOM files and then transferred to 3-matic Version Medical 17 (Materialise Leuven Belgium) to define the area of the implant that will be taken for micro-CT scanning.

The specimens were scanned by using a high-resolution desktop micro-CT system (Bruker Skyscan 1275, Kontich, Belgium). The scanning parameters were as follows: 100 kVp, 120 mA, 0.5 mm of Al or Cu light-tight filter, 15.2 μm pixel size, and rotation at 0.2 step. In order to reduce the occurrence of ring artifacts, the detector underwent an air calibration before each scanning process. The sample underwent a complete rotation of 360° over a time frame of 5 min. Additional configurations encompassed beam hardening correction and the input of ideal contrast thresholds in accordance with the manufacturer’s guidelines, relying on prior scanning and reconstruction of each specimen.

The NRecon software (version 1.6.10.5, SkyScan, Kontich, Belgium) and CTAn (version 1.19.11.1, SkyScan) were employed to visualize and quantitatively measure the samples. The modified algorithm described by Feldkamp et al.^35^ was utilized to acquire axial, two-dimensional (2D) 1000 × 1000-pixel images. The reconstruction parameters were set as follows: ring artifact correction and smoothness were both set to 0, while the beam artifact correction was set at 40%. The NRecon program (Skyscan, Kontich, Belgium) was utilized to rebuild the scanner’s pictures, resulting in 2D slices of the specimen. A total of 1023 cross-sectional pictures were reconstructed from the whole volume. In addition, the CTAn program from Skyscan in Aartselaar, Belgium, was utilized for the 3D volumetric visualization, analysis, and measurement of the specimen’s volume using micro-CT. The reconstructions were performed on a 21.3 in. flat-panel color-active matrix thin-film transistor (TFT) medical display (NEC MultiSync MD215MG, Munich, Germany) with a resolution of 2048 × 2560 at 75 Hz and a dot pitch of 0.17 mm. The display was operated at 11.9 bits. The reconstructed images underwent further processing in Skyscan CTVox software (version 3.3.1, Skyscan, Kontich, Belgium) to enhance their display.

Following the rebuilding process, the specific region of interest (ROI) was delineated to include the complete implant inside the sample using CTAn software. This program was utilized to examine the 3D microstructure of newly developed bones. In order to differentiate the recently developed bones from the skull, it is necessary to establish an appropriate threshold. This threshold value serves as the minimum boundary between gray values of 80 and 255, while the upper limit is determined based on the brightness spectrum that represents the greatest density value of the bones. To figure out the new bone in 3D volumes, the original grayscale pictures were processed using the global threshold approach^35^ and a Gaussian low-pass filter to get rid of the noise. After the thresholding operation, the resulting image consists of only black and white pixels. Subsequently, an ROI was selected for each individual slice to encompass a singular item completely, facilitating the computation of new bone tissue. The measurements of the new bone included the following structural parameters: tissue volume (TV), bone volume (BV), percent bone volume (BV/TV; %), trabecular thickness (mm), and bone surface/bone volume ratio (1/mm).

### 2.4 Histopathological Analysis

The samples were fixed for 48 h in neutral buffered formalin and then decalcified in ethylenediaminetetraacetic acid (EDTA) and hydrochloric acid solution (Biocal C, RRDC3-E, Atom Scientific Ltd.) for 3 days at 37 °C with daily solution replacement. Samples were then dehydrated in a graded series of ethanol (0–100%), cleared with xylene, and subsequently embedded in paraffin. Three serial 5 μm sections were collected from each sample and stained with hematoxylin and eosin (H&E, Merck) and Masson’s trichrome (Merck) for histopathological evaluation. The specimens were then examined under a light microscope (Olympus BX51) in a blinded manner and recorded with an optical microscope (Olympus DP71).

A quantitative grading scale (0–4) (Supporting Information, Table S1) was used to score the slides collected from each sample for (1) hard tissue response at the bone–implant interface and (2) quantity of bone formation within the defect.^36^ An overall score for each group was determined by averaging the issued scores for at least 6 samples.

### 2.5 Immunofluorescence Staining

Sections were obtained as described above and incubated with phosphate-buffered saline (PBS) containing 0.1% Tween20 and 1% bovine serum albumin (BSA) for 2 h at 4 °C to prevent nonspecific binding and permeabilize cell membranes. Specimens were then incubated with primary antibodies for osteopontin (1:500) (antiosteopontin antibody, Abcam ab8448, mouse) and CD31 (1:1000) (anti-CD31 antibody, Abcam ab182981, rabbit) diluted in PBS containing 1% BSA overnight at 4 °C. Afterward, sections were incubated with secondary antibodies Alexa Fluor 488-conjugated goat anti-mouse IgG (1:200) and Alexa Fluor 595-conjugated goat anti-rabbit IgG (1:200) for 1 h at room temperature. Nuclei were counterlabeled with 4′,6-diamidino-2-phenylindole (DAPI) and visualized on a confocal laser scanning microscope (Leica, Germany).

### 2.6 Statistical Analysis

All quantitative results were expressed as means ± standard deviation (*n* > 3). Data were analyzed with statistically significant values defined as *p* < 0.05 based on one-way analysis of variance (ANOVA) followed by Tukey’s test for determination of the significance of the difference between different groups (*p* ≤ 0.05).

## 3 Results

### 3.1 Analysis of *In Vivo* Biodegradation

**Figure 2:**
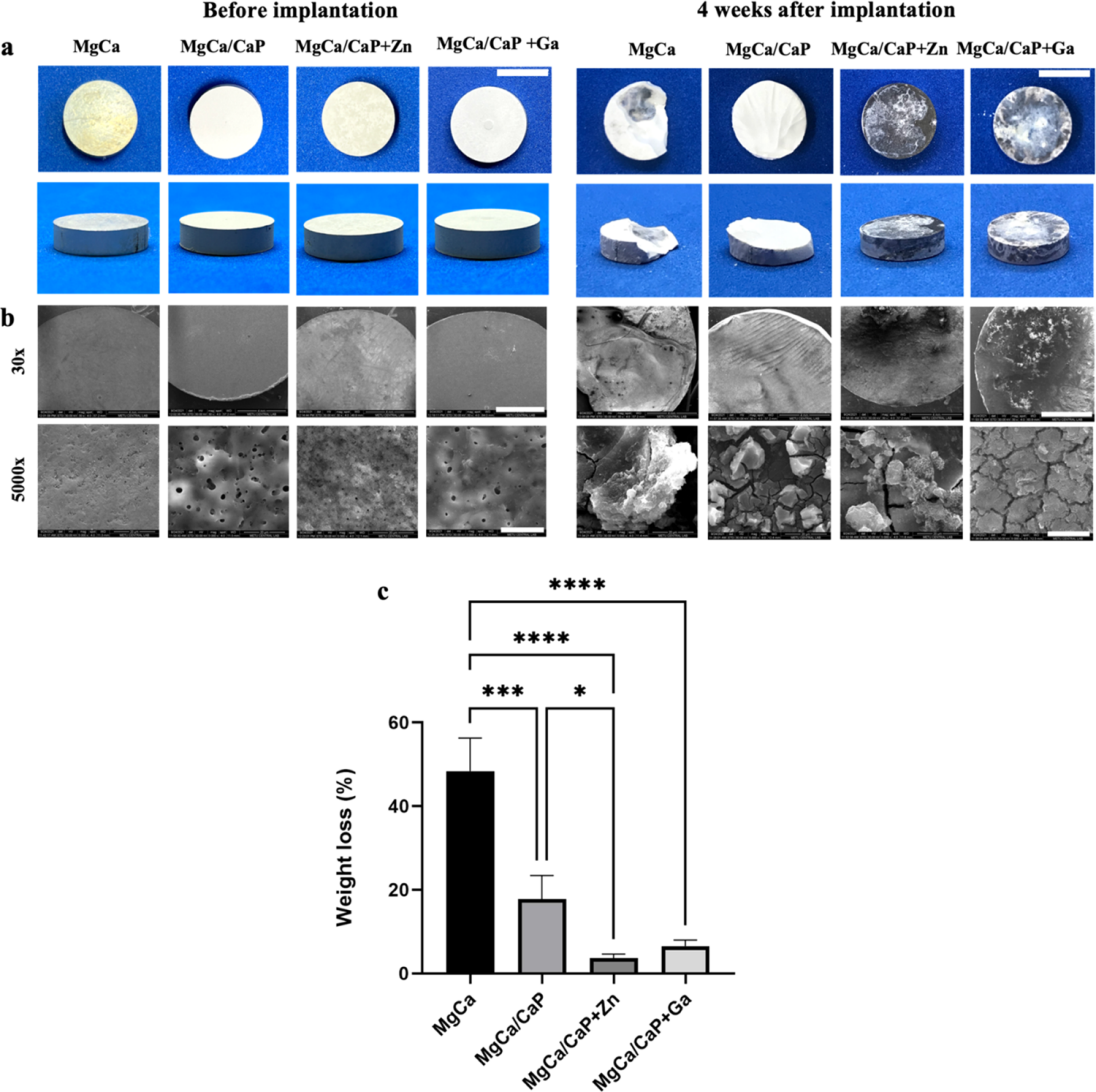
Degradation of the MgCa-based implants during 4 weeks of implantation within bone defects. (a) Gross observation of the implants before and after implantation (scale bar: 1 cm), (b) SEM micrographs of the implants before and after implantation (scale bars; Upper row: 8 mm (×30), Lower row: 40 μm (×5000)). (c) Weight loss (%) of the MgCa-based implants at 4 weeks after implantation (*p < 0.01, ***p = 0.0001, ****p < 0.00001).

MgCa-based implants were removed from the bone defects, and their biodegradation during 4 weeks of implantation was studied. It was observed by gross observation that although the implants have similar size and topography prior to implantation, they quite differ after 4 weeks *in vivo*, mainly based on the presence and/or absence of the CaP surface modification (Figure 2a). Uncoated MgCa implants lost their structural integrity the most, followed by apparent surface erosion of the MgCa/CaP implants. No macroscopically pronounced degradation was noted in the MgCa/CaP + Zn and MgCa/CaP + Ga implants.

The alterations on the surfaces of the implants after 4 weeks of implantation were investigated by SEM in comparison to their morphology prior to implantation (Figure 2b). It was observed that MgCa implants had smooth surface topography, where CaP coating (undoped and doped with Zn and Ga) introduced several levels of homogeneous surface roughness to the implants prior to implantation. After 4 weeks of implantation within the bone defects, it was observed that the surfaces of all of the implants deviated from their original topography to a great extent. The surface of MgCa implants appeared irregular, where implants coated with CaP (undoped and doped with Zn and Ga) exhibited crack formation on the surface.

The extent of biodegradation of the implants was also characterized by measuring their weight loss % gravimetrically (Figure 2c). The weight loss of the MgCa implant was ca. 50% during 4 weeks of implantation. This value was lowered down to ca. 15% when CaP surface treatment was available on the implant (MgCa/CaP). Moreover, the presence of Zn and Ga within the CaP coating further reduced the degradation profile. All of these changes were found to be statistically significant, indicating that MgCa implants degraded and deteriorated to a great extent as compared to the CaP-coated (undoped and doped with Zn or Ga) counterparts.

### 3.2 Radiological Investigation of the Implant Site

**Figure 3:**
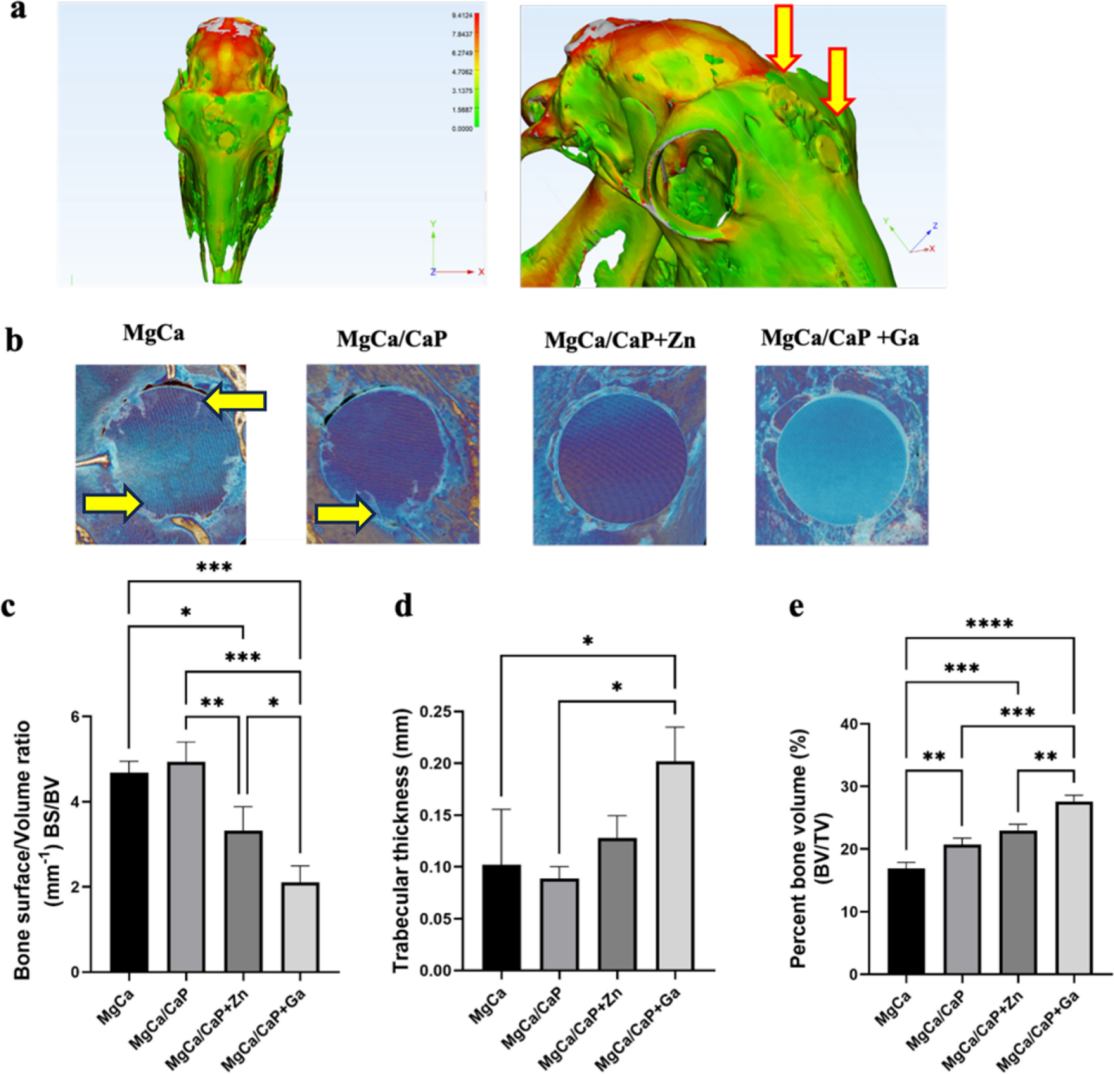
Radiological evaluation of the implant site with CBCT and micro-CT after 4 weeks of implantation. (a) Demonstration of the general placement site of implants and their integration with bone with CBCT. Yellow arrows show the implants. (b) Micro-CT scans of the defect regions; (c) bone surface/volume ratio (BS/BV; 1/mm); (d) trabecular thickness (mm); (e) bone volume (%) (bone volume/total volume) calculated from the micro-CT analysis results (*p < 0.01, **p < 0.001, ***p < 0.0001, ****p < 0.00001).

CBCT scans revealed the presence of the implants within the skull defects and their integration with the bone tissue at 4 weeks after implantation (Figure 3a). According to the micro-CT scans, there was no visual significant difference between the implant–bone integration profiles among implant groups, while a more direct contact and union was noted in MgCa/CaP + Zn and MgCa/CaP + Ga implants as compared to the others (Figure 3b).

In the quantitative analysis of the radiological images, it was observed that bone quality was higher in the MgCa and MgCa/CaP implants, as shown by the bone surface/bone volume ratio (Figure 3c). Although no significant difference was observed between the MgCa, MgCa/CaP, and MgCa/CaP + Zn implants in trabecular thickness, MgCa/CaP + Ga was observed to be statistically significantly higher than the other samples, indicating thicker bone regeneration within the defect (Figure 3d). Bone volume to total tissue volume ratio was observed to be the highest in MgCa/CaP + Ga implants (Figure 3e), indicating more bone formation within the defect area and the presence of less unorganized fibrous tissue.

### 3.3 Histopathological Analysis of Bone–Implant Interface and Bone Regeneration within the Defect

**Figure 4:**
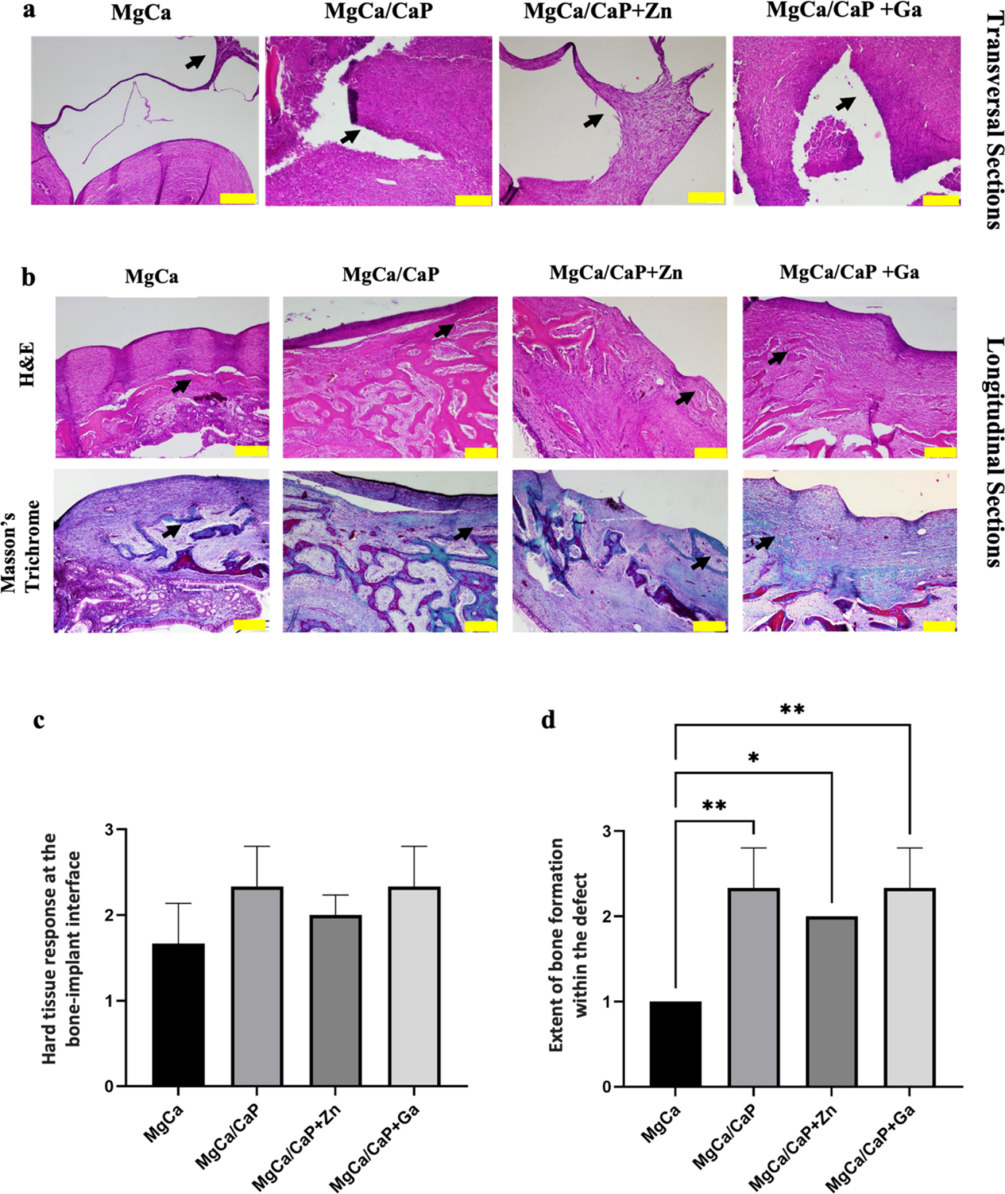
Histopathological examination of the bone tissue at 4 weeks after implantation with MgCa-based implants. (a) H&E staining of the bone–implant interface in transversal sections. New bone formation (black arrows) and connective tissue capsule of various thickenings lining the interface (scale bar: 200 μm). (b) H&E and Masson’s trichrome staining of the defect site in longitudinal sections. Black arrows show new bone formation (scale bar: 200 μm). Results of histopathological scoring for (c) hard tissue response at the bone–implant interface and (d) extent of bone formation within the defect (*p < 0.01, **p < 0.001).

Hematoxylin and eosin (H&E) and Masson’s trichrome staining of the bone–implant interface showed differences among groups. New bone formation and connective tissue capsules of various thicknesses lining the interface were observed in all implant groups (Figure 4a,b). Evaluation of the samples for hard tissue response at the bone–implant interface showed no significant difference between the groups (*p* > 0.1) (Figure 4c). All types of implants were surrounded by fibrous tissue capsules composed of organized connective tissue cells. Newly formed bone trabeculae were generally separated from the implant interface by an intermediate fibrous layer. However, a slightly higher direct bone–implant contact was observed in the MgCa/CaP implant and MgCa/CaP + Zn implant compared to others (Figure 4c). In these 2 implants, few lacunae of newly formed bone were observed in direct contact with the implant interface in a few areas.

The extent of bone formation in the defects was lower in the presence of MgCa implants as compared to the others with surface modification (*p* < 0.01) (Figure 4d). A larger number of newly born trabecular bones were filling the defect surface with CaP-coated implants in general compared to uncoated implants.

Despite the active bone formation, connective tissue proliferation, and neovascularization surrounding the defect area, prominent necro-inflammatory reaction was observed in both MgCa/CaP and MgCa/CaP + Ga implants. This inflammatory reaction was mainly composed of plasma cells, lymphocytes, and macrophages with few neutrophils accompanied by necrosis. In comparison, a very sparse to absent inflammatory reaction was observed in MgCa/CaP + Zn implants.

### 3.4 Immunohistochemical Analysis for Bone Regeneration and Neovascularization at the Implant Site

**Figure 5:**
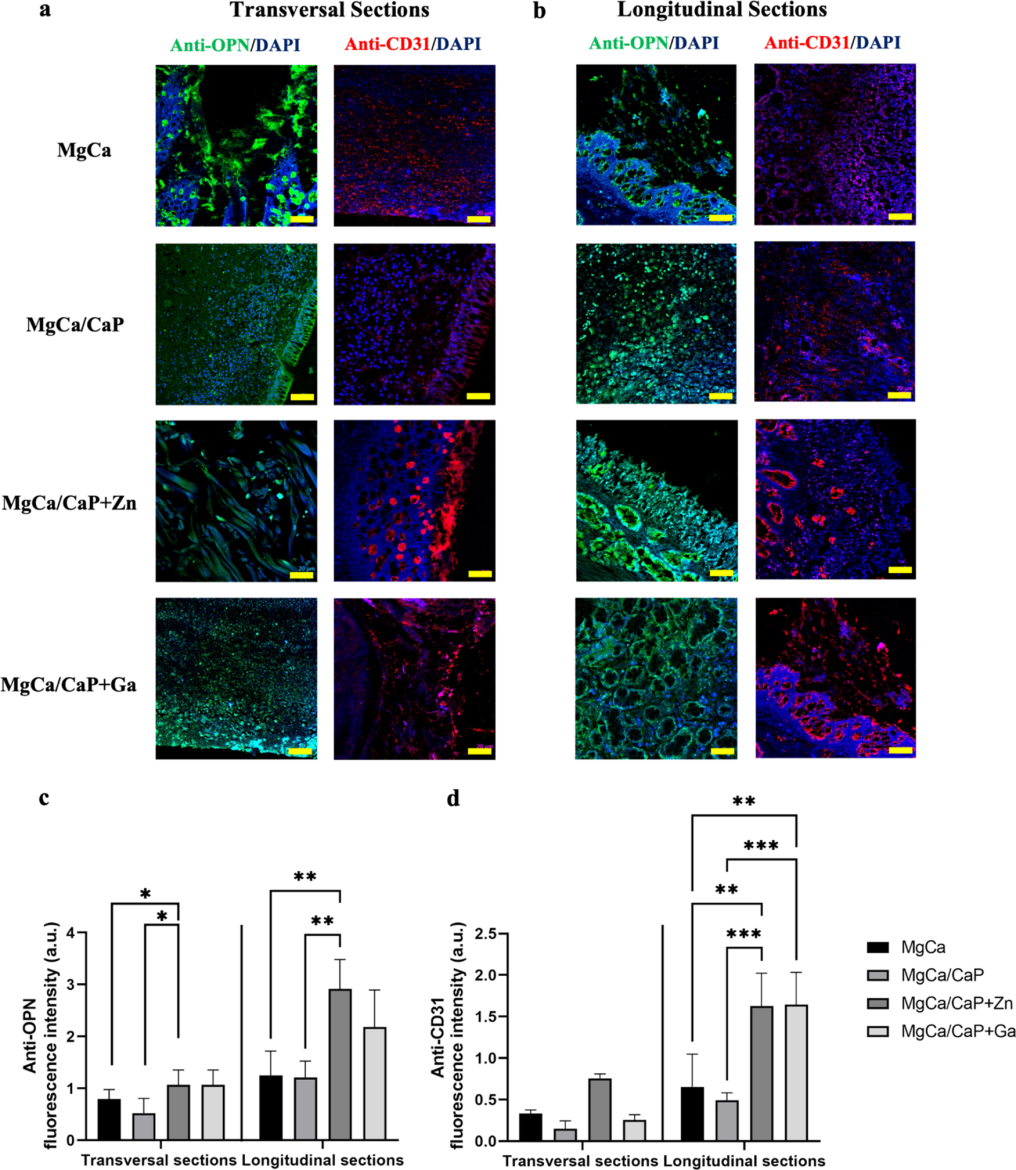
Immunohistochemical analysis of the bone regeneration and neovascularization at the defect sites stained for osteopontin (OPN) (green) and CD31 (red). Immunofluorescence staining images of (a) transversal (scale bar: 100 μm) and (b) longitudinal (scale bar: 400 μm) sections. Staining intensity in the images was quantified using ImageJ. (c) Quantification of OPN staining at the transversal and longitudinal sections; (d) quantification of CD31 staining at the transversal and longitudinal sections (*p > 0.01, **p > 0.0030, ***p < 0.0005, ****p < 0.0001).

Immunofluorescence staining for osteopontin (OPN) and CD31 was performed to identify the newly formed bone and vascular structures at the defect site, respectively. It was observed in both transversal and longitudinal sections that MgCa/CaP + Zn and MgCa/CaP + Ga implants provided a higher amount of OPN-positive staining as compared to the other groups (Figure 5a,b). This trend was statistically significantly higher in the longitudinal sections compared to the transversal sections (Figure 5c,d). OPN intensity was not statistically different in the transversal sections although Zn- and Ga-doped CaP coating provided higher positive staining. OPN intensity was statistically significantly higher for MgCa/CaP + Zn implants as compared to the others (*p* < 0.01).

CD31 staining was higher in MgCa/CaP implants doped with Zn and Ga (Figure 5a,b). Lumen formation was evident in the longitudinal sections of the defects implanted with MgCa/CaP + Zn. CD31 staining provided the highest intensity values for the MgCa/CaP + Zn implants in both longitudinal and transversal sections compared to the MgCa and MgCa/CaP implants (*p* < 0.01) (Figure 5e,f). While the CD31 staining was statistically significantly higher in MgCa/CaP + Zn compared to MgCa/CaP + Ga in the transversal sections (*p* < 0.0001), this difference was not significant in the longitudinal sections.

## 4 Discussion

### 4.1 Biodegradation of MgCa Implants within Bone Defects Are Fine-Tuned Via Surface Modification

Implants are widely used for orthopedic and dental applications, including fixing fractures, repairing nonunions, and obtaining joint arthrodesis and arthroplasty, spinal reconstruction, and soft tissue anchorage. These implants are frequently made of metals since there is no current alternative to achieve the strength and structural stability required for load-bearing applications. The use of polymers and their composites is generally limited to non-load-bearing applications such as the craniofacial area.

The use of nondegradable metallic implants necessitates secondary surgeries for implant removal after the fracture is healed. Drawbacks also include the occurrence of osteoporosis in the underlying bone due to the stress-shielding effect resulting from the mismatch in the bone and metal material stiffness. Such cases fueled research for the development of biodegradable metallic implants. Mg alloys are increasingly being investigated as biodegradable metallic implants, along with Zn, Al, Fe, etc., based alloys and their composites.^37−41^ However, the fast corrosion rate and structural instability are viewed as the current limitation for their clinical use.^41^

There are several mechanisms of Mg implant degradation *in vivo.*(42,43) These include electrochemical corrosion, hydroxide and hydrogen formation, and chloride attack. The hydrogen that evolved can accumulate as gas bubbles within the defect area, causing serious clinical problems. Alloying with Al, Zn, or Ca elements enhances the mechanical properties and corrosion resistance, whereas alloying with rare earth elements like yttrium (Y) and gadolinium (Gd) refines the microstructure and improves the corrosion resistance of Mg implants. Moreover, modification of the surface is being accepted as an effective strategy to control the physical and biological properties of implants.^44^ These studies include surface treatment of implants with anodization to create a protective oxide layer to slow down corrosion or chemical conversion coatings that form protective layers like magnesium phosphate. Coating of implant surfaces with biodegradable polymers (e.g., poly(lactic acid)) is also shown to be an effective strategy to act as a barrier to corrosion. Surface treatment of implants is also performed with compounds having biological activity in order to enhance compatibility with the surrounding tissue.^45,46^ There are several reports in the literature on the surface modification of Mg-based implants with bioactive coatings. For example, in the study of Gao et al.,^47^ Mg-based implants were produced and coated with CaP without any ion doping, and delayed degradation was observed. We have also previously reported that the *in vitro* corrosion and degradation rate of the MgCa alloys can be effectively diminished by surface treatment via microarc oxidation of CaP either doped or undoped with Zn.^26^ Here, a similar observation was obtained in the orthotopic implantation model of these implants (Figure 1), as well. When MgCa implants were surface treated with CaP and implanted within sheep cranial defects, the degradation of the implants was decreased significantly compared to that of the untreated MgCa (Figure 2). Bare MgCa implants lost almost half of their weight, and their structural integrity was not preserved during 4 weeks of implantation within the bone defects. This property was improved significantly by surface treatment with CaP and Zn-/Ga-doped CaP. This finding is consistent with the literature, where a more controlled degradation rate was reported by surface modification of Mg-based alloys.^29,31,32^

Biodegradation of the metal implants is also related to their corrosion characteristics under physiological conditions. The corrosion rates of Zn-doped Mg implants were reported to be in between 0.96 and 1.72 mm/year under *in vitro* conditions. This fast corrosion rate was significantly lowered (to 0.38 mm/year) in an *in vivo* study.^48^ Our previous study shows that Zn-doped coatings improve corrosion resistance compared to the uncoated MgCa substrates.^28^ Therefore, this property also adds to the fine-tuning of the biodegradation behavior. Animal experimentation with rats and rabbits provides very useful information on the developmental stages and biocompatibility of medical devices, including implants, although data produced on large animal models (such as sheep) provide a much better understanding of the potential clinical outcomes. Therefore, we believe that this study is important toward the clinical translation of Mg-based implants.

### 4.2 Bone Regeneration within the Defects Is Enhanced by MgCa/CaP Implants Doped with Zn and Ga

We have studied bone regeneration within the defects implanted with MgCa-based implants by radiological, histopathological, and immunohistochemical analysis (Figures 3–5). Quantitative assessment of the micro-CT images revealed that the trabecular thickness within the defects was significantly higher in the case of MgCa/CaP + Ga implants compared to others, indicating that bone regeneration was more throughout along all edges and along the thickness of the defect. Moreover, bone volume/total volume within the defect was also the highest for MgCa/CaP + Ga implants, again corresponding to more uniform bone regeneration (Figure 3).

Results in Figure 4 showed that while CaP, CaP + Ga, and CaP + Zn coating of the implants improved bone regeneration and differentiation of osteoblasts, CaP + Zn coating was remarkably accompanied by less adverse effects with a minimal inflammatory reaction and a prominent bone regeneration. The CaP coating on the MgCa implants improved peri-implant bone formation by promoting the proliferation of osteoblasts and connective tissue cells. Quantitative analysis of the histopathological sections stained with H&E and Masson’s trichrome suggested that the MgCa implant exhibited enhanced bone regeneration capacity when modified with CaP coating and otherwise was rich with mostly immature connective tissue cells (Figure 4). While MgCa/CaP- and MgCa/CaP + Ga-coated implants resulted in larger amounts of inflammatory cells in some defect areas, the defects implanted with MgCa/CaP + Zn implants were free of inflammatory cells. MgCa/CaP + Zn implant specially contained well-organized connective tissue cells and neovascularization. These results suggested that the bone regeneration potential of MgCa implants can be greatly enhanced by surface modification with Zn-doped CaP coating, as Zn is a well-known agent to induce osteogenesis.^49^

There are only a few studies in the literature that report the osteogenic effects of Mg-based biodegradable implants (screws) in sheep models (implanted in the tibia). In the studies of Marek et al.,^50,51^ Mg-based alloy ZX00 (Mg < 0.5 Zn < 0.5 Ca, in wt %) was implanted in sheep tibia, and the osteointegration potential as well as biodegradation were studied radiologically and histopathologically. In another study,^52^ Mg-0.45Zn-0.45Ca (ZX00) screws were implanted in sheep tibia again, and the osteogenic potential as well as the degradation profile were assessed. To the best of our knowledge, this study is novel in terms of investigating the vascularized bone regeneration (osteogenic and angiogenic) potential of Mg-based implants in a sheep cranial defect model, which is important to assess the bone–implant interactions free of biomechanical stresses. Results in Figure 5 suggested that both osteogenic and angiogenic markers (OPN and CD31) stained significantly higher in longitudinal sections corresponding to the cross section of the defects, leading to enhanced vascularized bone regeneration in the presence of Ga- and Zn-doped CaP coatings on the MgCa implants. Therefore, the results of this study are important in underlying the bone–Mg-based alloy interactions as the sole determinant.

We have investigated the MgCa-based implants in a non-load-bearing bone implantation model and showed that the incorporation of Zn to the MgCa-based implants’ surface treated with CaP is an effective strategy to both control the degradation behavior of the implants and to enhance their osteogenic potential.

### 4.3 Neovascularization Is Initiated by the Zn-doped MgCa/CaP Implants within the Defect

Bone is a vascularized tissue, and the survival of the inherent cells relies on the density and architecture of this vascularization.^53^ Vascularization is therefore a key factor in the survival of bone graft materials and in the success of biodegradable implants. Its absence or inhibition will lead to necrotic cores within the bone tissue, which hinders the functionality of the regenerated tissue. Neovascularization within the bone grafts and biodegradable implants is also very important in terms of ensuring anastomosis with the vessels in the transplantation area after implantation, which will lead to viable and successful outcomes.^54−56^

Recent studies have focused on modifying Mg alloys to promote angiogenic responses, aiming to optimize their performance in implantable devices.^57^ Strategies include the incorporation of specific alloying elements, such as St or Zn, which have been shown to positively influence vascularization.^30,34^ In the study of Liu et al.,^32^ the effect of the implant consisting of Mg, Zn, and Mn was examined on the angiogenesis of human umbilical vein endothelial cells (HUVECs). It was reported that the extracts from the Mg–Zn–Mn alloy induce the angiogenesis of HUVECs. The direct contact of the cells with the implant was not studied. In another study, a Mg-based rod was implanted in a rat model, and the animals were treated with a calcitonin gene-related peptide (CGRP) receptor’s antagonist or a VEGF receptor-2 inhibitor. It was observed that the Mg implant significantly reduced the occurrence of osteonecrosis-like lesions and increased bone microstructural parameters and expression of vascular endothelial growth factor A (VEGFA).^58^ These studies paved the way for further investigation of the effect of Mg-based implants on their proangiogenic effects and potential for regeneration of vascularized bone tissue in larger animal models. In the present study, Mg-based implants, which have modified surfaces with Zn- or Ga-doped CaP, were implanted in sheep cranial defects, and we observed similar positive results on angiogenesis, which was shown by the presence of enhanced positive CD31 staining in the presence of MgCa/CaP + Zn implants. It is important to note that doping Zn within CaP coatings on MgCa implants has an important positive effect on angiogenesis (Figure 5), depicting the vascularized bone regeneration potential (both osteogenic and angiogenic effect) of Mg-based implants in a sheep cranial defect model.

## 5 Conclusions

Biodegradable metals are gaining increased attention due to their favorable properties such as eliminating the need for secondary surgeries for implant removal while having suitable mechanical properties as compared to conventional biodegradable polymers. Their fast degradation and corrosion rate are the current limiting step in their use. In this work, we report on the control of the degradation rate of MgCa alloys, surface modified with CaP (either doped or undoped with Zn and Ga) in a sheep cranium implantation model. Our results also showed that the MgCa implants’ surface modified with Zn containing CaP results in enhanced bone formation and neovascularization within the defect at 4 weeks after implantation. Therefore, an effective strategy is being validated in an *in vivo*, large animal model, an important step toward the clinical use of Mg-based alloys as biodegradable implants.

## Supporting Information Available

Guide table for quantitative histopathological analysis (PDF)
