# Supplementary material for: MgCa-Based Alloys Modified with Zn- and Ga-Doped CaP Coatings Lead to Controlled Degradation and Enhanced Bone Formation in a Sheep Cranium Defect Model
Source: ACS Biomater Sci Eng. 2024 Jun 14;10(7):4452–62. doi: 10.1021/acsbiomaterials.4c00358 (PMC11234335; doi:10.1021/acsbiomaterials.4c00358)
Supplement: Supplementary file 1 — ab4c00358_si_001.pdf [file ab4c00358_si_001.pdf]

MgCa-Based Alloys Modified with Zn- and Ga-Doped CaP Coatings Lead to Controlled Degradation and Enhanced Bone Formation in a Sheep Cranium Defect Model

Seyda Gokyer[1], Yanad Abou Monsef[2], Senem Buyuksungur[3], Jurgen Schmidt[4], Alina Vladescu (Dragomir)[5,6], Sencer Uygur[7], Cagdas Oto[8,9], Kaan Orhan[9,10], Vasif Hasirci[3,11,12,13], Nesrin Hasirci[3,14,15*] Pinar Yilgor[1,9*]

[1] Ankara University, Department of Biomedical Engineering, Ankara 06830, Turkey

[2] National Veterinary School of Toulouse, Anatomic Pathology Department, Toulouse 31300, France

[3] Middle East Technical University (METU), BIOMATEN, Center of Excellence in Biomaterials and Tissue Engineering, Ankara 06800, Turkey

[4] INNOVENT e.V. Technologieentwicklung, Gruppenleiter Elektrochemie, Prüssingstraße 27b, 07745 Jena, Germany

[5] National Institute of R&D for Optoelectronics - INOE 2000, 409 Atomistilor St., Magurele 77125, Romania

[6] Research School of Chemistry & Applied Biomedical Sciences, National Research Tomsk Polytechnic University, Tomsk 634050, Russia

Ankara University, [7] Faculty of Veterinary Medicine Department of Surgery, [8] Faculty of Veterinary Medicine Department of Anatomy, [9] Medical Design Research and Application Center MEDITAM, [10] Faculty of Dentistry Department of Dentomaxillofacial Radiology, Ankara 06100, Turkey

Acibadem Mehmet Ali Aydinlar University, [11] Department of Biomedical Engineering, [12] Graduate Department of Biomaterials, [13] Biomaterials Center, Istanbul 34752, Turkey,

[14] METU Department of Chemistry, Ankara 06800, Turkey

[15] Near East University Tissue Engineering and Biomaterials Research Center, Nicosia, TRNC, Mersin 10, 99138, Turkey

***Correspondence to:** Prof. Nesrin Hasirci (nhasirci@metu.edu.tr) and Prof. Pinar Yilgor Huri (E-mail: phuri@ankara.edu.tr)

Supplementary Table 1. Guide table for quantitative histopathological analysis.

**Hard Tissue Response at the Bone-Implant Interface**		**Prevalence of Bone Formation within the Defect**	
Definition	**Score**	***Definition***	**Score**
Direct bone-implant contact without soft tissue interface	4	75-100% of the defect consists of bone	4
Remodeling of lacunae with osteoblasts and/or osteoclasts on the surface	3	50-74% of the defect consists of bone	3
Most of the implant is surrounded by a fibrous capsule	2	25-49% of the defect consists of bone	2
Disorganized fibrous tissue (most of the tissue is not organized to form a capsule)	1	1-24% of the defect consists of bone	1
Inflammation characterized by the presence of dense inflammatory cells and poorly organized tissue	0	0% of the defect consists of bone or there is only some bone around the edges	0
